# Association between Serum Vitamin D Status and Circadian Syndrome: A Cross-Sectional Study

**DOI:** 10.3390/nu16132111

**Published:** 2024-07-02

**Authors:** Ahmed Arabi, Dima Nasrallah, Sara Mohsen, Lana Abugharbieh, Dana Al-Hashimi, Shaikha AlMass, Shahd Albasti, Saeed A. Al-Ajmi, Muhammad Naseem Khan, Susu M. Zughaier

**Affiliations:** 1 College of Medicine, QU Health, Qatar University, Doha P.O. Box 2713, Qatar; 2 Department of Population Medicine, College of Medicine, QU Health, Qatar University, Doha P.O. Box 2713, Qatar; 3 Department of Basic Medical Sciences, College of Medicine, QU Health, Qatar University, Doha P.O. Box 2713, Qatar

**Keywords:** Vitamin D, Circadian Syndrome (CircS), Metabolic Syndrome (MetS), Type 2 diabetes, NHANES

## Abstract

Background: Circadian Syndrome (CircS) encompasses cardiometabolic risk factors and comorbidities, indicating an elevated susceptibility to cardiovascular disease and type 2 diabetes. Methods: This cross-sectional study aimed to investigate the association between vitamin D levels and each of the following: CircS, metabolic syndrome (MetS), and the individual components of CircS. Data from 14,907 adults who participated in the National Health and Nutrition Examination Survey (NHANES) between 2007 and 2018 were utilized. CircS was defined based on MetS components, alongside depression, short sleep, and non-alcoholic fatty liver disease (NAFLD). Results: Our results indicated that low vitamin D levels exhibited meaningful associations with CircS, with vitamin D deficiency and inadequacy demonstrating 2.21-fold (95% CI 1.78–2.74, *p* < 0.001) and 1.33-fold (95% CI 1.14–1.54, *p* < 0.001) increases in CircS odds, respectively. The association between vitamin D deficiency and CircS was stronger than that with MetS. Additionally, a dose-response gradient in odds of CircS components, particularly with short sleep duration, was noted as serum vitamin D levels decreased. Conclusions: our findings highlight a significant association between low serum vitamin D levels and CircS and its components, particularly with short sleep. This suggests a potentially pivotal role of vitamin D in the pathogenesis of Circadian syndrome.

## 1. Introduction

Circadian syndrome (CircS) is a novel comprehensive concept that has been introduced to collectively address a cluster of cardiometabolic risk factors along with additional comorbidities within a singular, unified syndrome that increases susceptibility to cardiovascular disease (CVD) and type 2 diabetes mellitus (T2DM) [1]. This concept originally stemmed from the foundational metabolic syndrome (MetS), which encompasses five components, including central obesity, elevated fasting plasma glucose (FPG), dyslipidemia defined by high triglycerides (TG) and/or low high-density lipoprotein cholesterol (HDL), and elevated blood pressure [2]. The traditional MetS was modified by adding three additional comorbidities, including sleep disturbance, depression, and non-alcoholic fatty liver disease (NAFLD), forming the recently established CircS [1]. This modification was driven by the mounting evidence pointing towards the tight association of circadian rhythm with metabolic health and homeostatic processes in the body [3]. In fact, it has been proposed that the so-called “circadian clock”, situated in the suprachiasmatic nucleus (SCN) in the hypothalamus, regulates metabolism by controlling gene expression, hormone release, activity patterns, and energy expenditure [3,4]. Accordingly, large body of research has established an association between circadian disruption and the various components of CircS [5,6,7,8,9,10]. Additionally, CircS is a superior predictor of CVD compared to MetS [1].

Given the profound significance of CircS in predicting CVD and T2DM, it has become imperative to understand its underlying causes and risk factors. Deficiency of vitamin D, a lipid-soluble vitamin existing in two forms, vitamin D2 (ergocalciferol) and vitamin D3 (cholecalciferol) [11], constitutes a major potential cause of CircS, given not only its well-established association with MetS [12,13], but also its link to sleep disturbances [14,15]. Vitamin D deficiency has also been shown to be associated with metabolic diseases, such as T2DM [16] and CVD [17]. This finding is further substantiated by the fact that beyond its renowned role in calcium homeostasis, calcitriol, the hormonally active metabolite of vitamin D, contributes to the regulation of glucose and lipid metabolism. Calcitriol influences glucose metabolism through various mechanisms, including the upregulation of the sirtuin 1/insulin receptor substrate-1/glucose transport type 4 (SIRT1/IRS1/GLUT-4) signaling pathway and the modulation of intracellular calcium levels, impacting insulin secretion [18,19]. Moreover, calcitriol exerts a significant effect on lipid metabolism through several proposed mechanisms, including the enhancement of bile salt production and the reduction of lecithin-cholesterol acyltransferase activity, both of which are crucial for reversing cholesterol transport [20].

Due to its recent emergence, there is a noticeable absence of discussion on CircS in literature. Therefore, in this research, we aim to explore the association between serum vitamin D levels and CircS status among adults enrolled in the National Health and Nutrition Examination Survey (NHANES) in the period spanning from 2007 to 2018 as a primary outcome. For the secondary analyses, we further compare this association with vitamin D’s association with MetS. Moreover, we investigate the association between vitamin D and the individual components of CircS. Taken together, this cross-sectional study provides valuable insight into the association of vitamin D deficiency with various cardiometabolic disturbances.

## 2. Materials and Methods

### 2.1. Study Design and Sample

NHANES run by the Centers for Disease Control and Prevention (CDC) is a cross-sectional survey aimed at evaluating the health and nutritional status of the United States’ population. NHANES ensures the representation of diverse demographics across all 50 states by utilizing a multistage probability sampling technique. Data collection methodologies include face-to-face or telephonic interviews, detailed questionnaires, laboratory analyses, and physical examinations [21]. Ethical oversight is maintained through approval by the National Center for Health Statistics Institutional Ethics Review Board, with written consent obtained from all participants. The survey data and methodologies are available for review and utilization at https://www.cdc.gov/nchs/nhanes/index.htm (accessed on 21 December 2023).

In this cross-sectional study, data from six NHANES cycles spanning from 2007 to 2018 with 59,842 participants was utilized. Individuals aged <18 years (23,262 participants) and those with incomplete data on serum vitamin D status (4200 participants) or CircS status (11,450 participants), as well as those with incomplete data on other covariates (6023 participants) were excluded from the analysis. 14,907 participants were included in the final analytical sample for the primary outcome analysis. Secondary outcome analyses, investigating the association between serum vitamin D status and individual CircS components, as well as between serum vitamin D status and MetS, were carried out on the subset of 32,380 participants aged ≥18 years with available data on serum vitamin D status. Participants with incomplete data on a specific CircS component or its associated covariates were excluded from the corresponding component’s analysis. Similarly, those with missing data on MetS or its covariates were excluded from the MetS analysis.

### 2.2. Exposure Measure: Vitamin D Status

A standardized and fully validated technique employing liquid chromatography-tandem mass spectrometry (LC-MS/MS) was utilized for the quantitative assessment of 25-hydroxyvitamin D3 (25OHD3), 3-epi-25-hydroxyvitamin D3 (epi-25OHD3), and 25-hydroxyvitamin D2 (25OHD2) in the serum of all eligible participants. Total serum 25(OH)D was defined as the combined concentrations of 25(OH)D3 and 25(OH)D2. According to the Institute of Medicine reference ranges, vitamin D status was classified into three groups: Adequacy (≥50 nmol/L = ≥20 ng/mL), inadequacy (30 nmol/L to <50 nmol/L = 12 ng/mL to <20 ng/mL), and deficiency (<30 nmol/L = <12 ng/mL) [22].

### 2.3. Outcome Measure: Circadian Syndrome Status

CircS was assessed based on eight components, with a cut-off of ≥5 components to confirm the diagnosis. These include (1) depression (Patient Health Questionnaire (PHQ-9) score of ≥10/27 [23]), (2) short sleep duration (self-reported <6 h/day [1]), and (3) non-alcoholic fatty liver disease (NAFLD). The remaining five components are the original MetS components [24], encompassing (4) elevated waist circumference (≥102 cm in males and ≥ 88 cm in females), (5) elevated blood pressure (Systolic ≥130 mm Hg and/or diastolic ≥85 mm Hg) or antihypertensive drug use in patients with hypertension, (6) low HDL-cholesterol (<40 mg/dL in men and <50 mg/dL in women) or drug treatment for reduced HDL-cholesterol, (7) high triglycerides (≥150 mg/dL) or drug treatment for elevated triglycerides, (8) and elevated fasting plasma glucose (FPG) (≥100 mg/dL), drug treatment for elevated glucose, or diagnosis of diabetes/prediabetes. 

NAFLD status was evaluated using the United States Fatty Liver Index (US-FLI). NAFLD was defined as having a US-FLI value of ≥30 in the absence of other liver disease etiologies, such as heavy alcohol consumption (>4 standard drinks per day for males and >3 standard drinks per day for females [25]), hepatitis B infection (HBsAg positive), or hepatitis C infection (HCV RNA positive).

### 2.4. Covariates

The following variables were included in the primary outcome analysis as covariates:

1. Self-reported demographic variables including age (years), race (Hispanic, white, black, and others/multi-racial), and socioeconomic status. Socioeconomic status was defined based on level of education and poverty income ratio (PIR). Education level was divided into three categories (below high school, high school, and graduate), while PIR was calculated by dividing a family’s income by the appropriate family poverty threshold that corresponds to the family’s size, year, and state. The resulting ratios were then categorized into six levels (PIR <1, 1–1.9, 2–2.9, 3–3.9, 4–4.9, and ≥5), in which higher values indicate greater financial stability.
2. Self-reported lifestyle variables, including dietary quality and physical activity. First, dietary quality was evaluated using the Healthy Eating Index-2020 (HEI-2020), which assesses the alignment of diet with the 2020–2025 Dietary Guidelines for Americans (DGA) [26]. The HEI-2020 comprises 13 components that evaluate the adequacy of food recommended in a healthy diet and the food that should be consumed in moderation. Each component is scored, contributing to an overall HEI-2020 score out of 100 points. Participants with scores within or above the 60th percentile were classified as having high dietary quality [27]. Second, physical activity, based on the Global Physical Activity Questionnaire (GPAQ), was assessed across five categories: vigorous-intensity work-related activity, moderate-intensity work-related activity, walking or bicycling for transportation, vigorous-intensity leisure-time activity, and moderate-intensity leisure-time activity. Each activity category was assigned a Metabolic Equivalent of Task (MET) score of 8, 4, 4, 8, and 4, respectively. MET hours per week were then estimated across all five categories and aggregated to derive an estimate of total physical activity with higher values indicating better physical activity.
3. Chronic disease variables including chronic kidney disease (CKD) status (self-reported) and liver cirrhosis status. For the diagnosis of liver cirrhosis, an Aspartate Aminotransferase (AST) to Platelet Ratio Index (APRI) score of >1 was utilized [28].

### 2.5. Statistical Analysis 

Categorical variables were presented as frequencies (N) and proportions (%). As for continuous variables, normality was first assessed using histograms and all variables were found to be non-normally distributed and thus, presented using medians and interquartile ranges (IQR). Group differences were tested using Pearson’s chi-squared test for categorical variables and Wilcoxon rank-sum test for continuous variables. Both Bar graphs and Line graphs were used to present data where appropriate. Odds ratios (OR) were generated using multivariable logistic regressions to assess the association between the exposure and outcome when adjusting for a set of covariates determined through Directed Acyclic Graphs (DAG) (Supplementary Figure S1). Both prognostic factors and confounders were adjusted for. A restricted cubic spline was utilized for variables that are non-linear with the outcome to achieve linearity. Goodness of fit was assessed using Receiver Operating Characteristic (ROC) curve and Area Under the Curve (AUC), whereas goodness of link was assessed using the *linktest* in Stata. 95% confidence intervals (CI) and *p*-Values were reported when appropriate. All statistical analysis was conducted using Stata version SE18 (Stata Corp., College Station, TX, USA).

## 3. Results

### 3.1. Baseline Characteristics

A total of 14,907 participants were included in the primary outcome analysis. Of those, 1341 (9.0%) participants were diagnosed with CircS. Baseline characteristics of all participants, stratified by CircS status, are shown in Table 1. CircS participants were older (60 years median age compared to 45 in the no CircS). Participants in both cohorts were predominantly white, comprising 44.0% of the no CircS group and 52.1% of the CircS group. The prevalence of vitamin D deficiency in the no CircS group is 7.40% compared to 10.50% in the CircS group. Notably, there were statistically significant differences between the two groups in all primary outcome analysis covariates. 

### 3.2. CircS Components Prevalence

**Figure 1:**
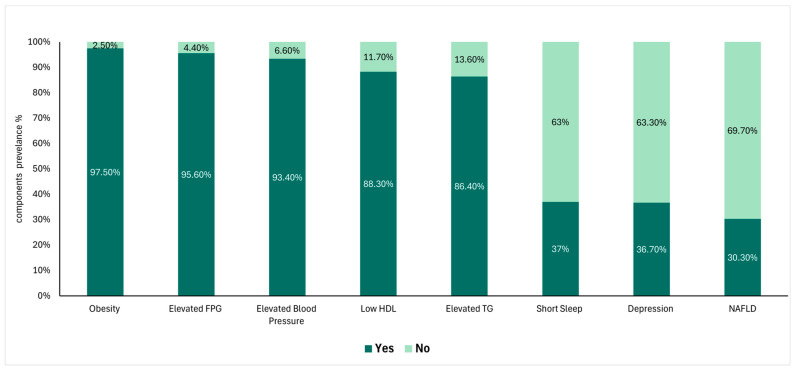
Stacked Bar Graph illustrating the prevalence of different CircS components among participants diagnosed with CircS (*n* = 1341).

Figure 1 illustrates the prevalence distribution of individual components comprising CircS. The vast majority of participants with CircS were found to be obese (97.5%). This was followed by having elevated FPG (95.6%), elevated blood pressure (93.4%), low HDL (88.30%), and elevated TG (86.4%). In contrast, less prevalent components included short sleep (37.0%), and depression (36.7%). The least abundant component was NAFLD with a prevalence of 30.3%.

### 3.3. Association between Vitamin D Status and CircS

Table 2 demonstrates the adjusted association between vitamin D status and CircS, investigated via multivariable logistic regression. The findings indicate a 2.21-fold increase in the odds of having CircS among individuals with vitamin D deficiency compared to those with vitamin D adequacy (95% CI 1.78–2.74, *p* < 0.001). Additionally, among participants with inadequate vitamin D levels, there was a 33% increase in the odds of CircS compared to those with adequate vitamin D levels (95% CI 1.14–1.54, *p* < 0.001). 

### 3.4. Association between Vitamin D Status and MetS

The adjusted association between vitamin D status and MetS was also analyzed using multivariable logistic regression, as illustrated in Table 3. Results suggest a 55% increase in the odds of having MetS among participants with deficient vitamin D levels compared to those with adequate levels (95% CI 1.34–1.79, *p* < 0.001). Furthermore, a 36% increase in the odds of having MetS was noted in participants with vitamin D inadequacy compared to those with adequacy (95% CI 1.24–1.49, *p* < 0.001). 

### 3.5. Association between Vitamin D Status and CircS Components

**Figure 2:**
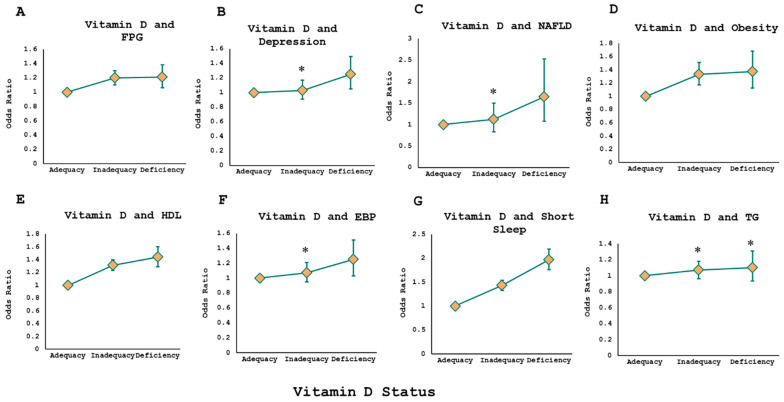
Dose-response curves showing the adjusted associations between serum vitamin D levels and different CircS components among included participants. Associations of vitamin D levels with elevated FPG (**A**), depression (**B**), NAFLD (**C**), obesity (**D**), low HDL (**E**), elevated blood pressure (EBP) (**F**), sleep (**G**), and elevated triglycerides (**H**). * Non-statistically significant findings. Detailed models adjusted for corresponding covariates are shown in Supplementary Tables S1–S8.

The association of vitamin D status with each of the eight components constituting CircS was assessed individually using multivariable logistic regressions. Supplementary Tables S1–S8 display those detailed regression analyses, including the different covariates adjusted for in each model. Out of the eight components, short sleep exhibited the most pronounced association with vitamin D deficiency, showing an almost 2-fold increase in odds compared to individuals with adequate vitamin D levels (OR 1.97, 95% CI 1.76–2.19, *p* < 0.001). Generally, a trend of rising odds was observed when moving from vitamin D adequacy to inadequacy and further to deficiency with each of the CircS components, as shown in Figure 2. The increase in odds when moving from inadequacy to deficiency was the greatest for short sleep, with a 54% increase in odds. NAFLD had the second-highest increase in odds with a 53% increase. However, other components had modest differences between odds comparing vitamin D inadequacy and deficiency.

## 4. Discussion

In this study, we investigated the association between serum vitamin D levels and several metabolic health and CVD predictors, namely CircS and its eight components as well as MetS, using a large representative sample from NHANES. The relationship between vitamin D status and CircS is multifaceted and intricate, influenced by numerous factors and key contributors, including age, race, socioeconomic status, diet quality, physical activity, liver cirrhosis, and chronic kidney disease (Supplementary Figure S1). Recognizing the substantial confounding and prognostic roles of the aforementioned factors, the analyses were adjusted accordingly. Our results indicate that low serum vitamin D levels are significantly associated with CircS status, as evidenced by a 2.21-fold and a 1.33-fold increase in the odds of CircS among individuals with vitamin D deficiency and inadequacy, respectively. Additionally, a progressive increase in the odds of having each component of CircS was observed as serum vitamin D levels declined from adequacy to inadequacy and further to deficiency, with the greatest association noted for sleep duration and NAFLD. A milder impact of vitamin D status on MetS was observed, with a 1.55-fold and a 1.36-fold increase in odds in cases of vitamin D deficiency and inadequacy, respectively.

Given the novelty of CircS, related research remains limited, yet existing literature explored the connection between serum vitamin D levels and its eight components individually. A systematic review investigating the interplay among vitamin D, MetS, and its components, unveiled a notable association between vitamin D and MetS [29]. As for the additional three comorbidities included in CircS, short sleep stood out in our analysis as the most strongly associated, with an almost 2-fold odds increase in vitamin D deficient individuals. This pronounced association observed between vitamin D levels and short sleep aligns with existing literature, as it has been established that vitamin D deficiency in children and adults increases the risk of sleeping difficulties, shorter sleep, and nocturnal awakenings [30,31]. Furthermore, consistent with our findings, a systematic review of observational studies underscored the relationship between low vitamin D levels and depression [32]. Concerning NAFLD, the association noted with vitamin D in our study is also consistent with prior studies. Bennouar et al. reported a strong association between severe vitamin D deficiency and NAFLD, with women (OR = 6.4, 95 CI 2.8–15, *p* < 0.001) having higher odds compared to men (OR = 5.8, 95% CI 1.9–17.7, *p* = 0.002) [33]. Additionally, higher vitamin D levels were found to be linked to lower risks of NAFLD in a dose-dependent manner, resulting in up to 50% reduction in NAFLD odds [34]. 

Our results also showed that vitamin D deficiency was more strongly associated with CircS than with MetS, despite CircS being defined by more stringent criteria, requiring at least five components compared to the three needed for MetS. This reflects the additional significance of including depression, NAFLD, and sleep duration in the definition of CircS. The disparity in odds ratios provides a possible explanation for the link between vitamin D, circadian system regulation, and cardiometabolic health indicators. These results first reinforce the evidence suggesting that circadian rhythm disruption is the driver behind all the various components of CircS, making it a more valuable reflector of cardiometabolic health than MetS [1]. Additionally, it consolidates both the evidence regarding the relationship between vitamin D levels and circadian system regulation [30,35], as well as vitamin D’s connection to MetS [36,37,38]. Taken together, we propose that vitamin D exerts significant influence on the underlying etiology of CircS and its subsequent complications, potentially through maintaining and regulating the circadian system. 

A plausible foundation of this hypothesis lies in the timing of sunlight exposure, particularly the onset and cessation of UV-B radiation during dawn and dusk, along with the corresponding rhythm of vitamin D synthesis. These elements serve as critical temporal cues for maintaining proper circadian rhythms [39]. Furthermore, although the precise mechanism remains understudied, evidence suggests that vitamin D may synchronize the expression of specific circadian clock genes, such as BMAL1 and Per2 [40,41]. Vitamin D is also implicated in the pathways involved in melatonin production, the hormone responsible for regulating human circadian rhythms and sleep [35]. Moreover, it has been documented that vitamin D receptors are present in brainstem regions crucial for the initiation and maintenance of sleep [30]. 

To the best of our knowledge, this study is the first to explore the relationship between serum vitamin D levels and Circadian Syndrome. A key strength of this study is the utilization of the NHANES database, renowned for its representation of the US general populace. Notably, our study included a substantially large sample of 14,907 participants, further enhancing the generalizability of our findings, albeit limited solely to the US population. Nevertheless, the study has a few limitations. First, NHANES adopts a cross-sectional design, precluding the drawing of inferences regarding directionality, causation, or temporal changes. In addition, despite adjustments made for potential confounders and prognostic factors, the presence of residual confounding cannot be entirely ruled out. Another limitation is that some NHANES variables rely on self-reported data rather than objective measurements. Finally, due to the absence of certain variables from NHANES, proxies such as US-FLI and APRI were utilized for diagnosing NAFLD and cirrhosis, respectively. 

Taken together, our study lays a foundation for subsequent research endeavors to probe the potential efficacy of vitamin D supplementation in mitigating and preventing CircS, its components, and subsequent complications among deficient individuals. Consequently, this could serve as the basis for the development and implementation of new guidelines and policies supporting the use of vitamin D supplementation in treating CircS, thereby preventing further complications. Additionally, longitudinal studies are imperative to establish causality and assess long-term effects of vitamin D status on the development and progression of CircS.

## 5. Conclusions

In conclusion, our findings indicate a significant association between vitamin D inadequacy and deficiency and CircS and its components, with short sleep showing the most prominent association. When comparing vitamin D’s relationship with MetS to its relationship with CircS, it becomes apparent that the association with CircS is notably more robust. These findings reinforce the association of vitamin D with both the circadian system and MetS, as well as the proposed link between circadian rhythm and cardiometabolic health. Collectively, these results underscore the potential utility of addressing cardiometabolic health through the lens of a single syndrome, CircS, and the plausible contribution of vitamin D in its preventative measures, thereby offering valuable insight into this field. However, further research is warranted to establish temporality and causality to validate these findings. 

## Figures and Tables

**Table 1:** Baseline characteristics of the study’s population categorized by CircS status (*n* = 14,907).

Characteristics	Categories	Circadian Syndrome N = 1341 N (%)	No Circadian Syndrome N = 13566 N (%)	*p*-Value
**Age in years, median (IQR)**		60.00 (49.00, 68.00)	45.00 (32.00, 61.00)	<0.001
**Sex**				
	Male	586 (43.7%)	6887 (50.8%)	<0.001
	Female	755 (56.3%)	6679 (49.2%)	
**Race**				
	Hispanic	352 (26.2%)	3156 (23.3%)	<0.001
	White	699 (52.1%)	5974 (44.0%)	
	Black	204 (15.2%)	2716 (20.0%)	
	Others/multi-racial	86 (6.4%)	1720 (12.7%)	
**Poverty income ratio**				
	PIR <1	353 (26.3%)	2616 (19.3%)	<0.001
	PIR 1–1.9	441 (32.9%)	3406 (25.1%)	
	PIR 2–2.9	196 (14.6%)	2127 (15.7%)	
	PIR 3–3.9	123 (9.2%)	1574 (11.6%)	
	PIR 4–4.9	83 (6.2%)	1152 (8.5%)	
	PIR ≥ 5	145 (10.8%)	2691 (19.8%)	
**Education level**				
	Below high school	417 (31.1%)	2656 (19.6%)	<0.001
	High school	749 (55.9%)	6954 (51.3%)	
	Graduate	175 (13.0%)	3956 (29.2%)	
**Vitamin D status**				
	Adequacy	899 (67.0%)	9571 (70.6%)	<0.001
	Inadequacy	301 (22.4%)	2994 (22.1%)	
	Deficiency	141 (10.5%)	1001 (7.4%)	
**Diet quality**				
	Low dietary quality	844 (62.9%)	7520 (55.4%)	<0.001
	High dietary quality	497 (37.1%)	6046 (44.6%)	
**Physical activity, median (IQR)**		6.0 (0.0, 40.0)	24.0 (4.0, 79.0)	<0.001
**Liver cirrhosis**				
	No cirrhosis	1299 (96.9%)	13,351 (98.4%)	<0.001
	Cirrhosis	42 (3.1%)	215 (1.6%)	
**Chronic kidney disease (CKD)**				
	Healthy	1213 (90.5%)	13,285 (97.9%)	<0.001
	CKD	128 (9.5%)	281 (2.1%)	

**Table 2:** Association between different serum vitamin D levels and CircS status among included participants (*n* = 14,907) ^1^.

Exposure	Categories	CircS Odds Ratio	*p*-Value	95% CI
Vitamin D Status				
	Adequacy	1		
	Inadequacy	1.33	<0.001	(1.14, 1.54)
	Deficiency	2.21	<0.001	(1.78, 2.74)

^1^ Model adjusted for age, race, poverty income ratio, education level, diet quality, physical activity, liver cirrhosis, and chronic kidney disease.

**Table 3:** Association between different serum vitamin D levels and MetS status among included participants (*n* = 14,621) ^1^.

Exposure	Categories	MetS Odds Ratio	*p*-Value	95% CI
Vitamin D Status				
	Adequacy	1		
	Inadequacy	1.36	<0.001	(1.24, 1.49)
	Deficiency	1.55	<0.001	(1.34, 1.79)

^1^ Model adjusted for age, race, poverty income ratio, education level, diet quality, physical activity, liver cirrhosis, and chronic kidney disease.

## Data Availability

Publicly available datasets were analyzed in this study. The dataset presented in this study can be found at https://www.cdc.gov/nchs/nhanes/index.htm (accessed on 21 December 2023).

## Supplementary Materials

The following supporting information can be downloaded at: https://www.mdpi.com/article/10.3390/nu16132111/s1, Figure S1: Directed Acyclic Graph (DAG) showing the association between vitamin D status with circadian syndrome and different covariates; Table S1: Association between serum vitamin D levels and obesity; Table S2: Association between serum vitamin D levels and short sleep; Table S3: Association between serum vitamin D levels and elevated FPG; Table S4: Association between serum vitamin D levels and low HDL; Table S5: Association between serum vitamin D levels and elevated TG; Table S6: Association between serum vitamin D levels and elevated blood pressure (EBP); Table S7: Association between serum vitamin D levels and NAFLD; Table S8: Association between serum vitamin D levels and Depression.

## References

[1] Akbar Z., Shi Z. (2023). Dietary Patterns and Circadian Syndrome among Adults Attending NHANES 2005–2016. Nutrients.

[2] Expert Panel on Detection, Evaluation, and Treatment of High Blood Cholesterol in Adults (2001). Executive Summary of the Third Report of the National Cholesterol Education Program (NCEP) Expert Panel on Detection, Evaluation, and Treatment of High Blood Cholesterol in Adults (Adult Treatment Panel III). JAMA.

[3] Panda S. (2019). The arrival of circadian medicine. Nat. Rev. Endocrinol..

[4] Orozco-Solis R., Sassone-Corsi P. (2014). Epigenetic control and the circadian clock: Linking metabolism to neuronal responses. Neuroscience.

[5] McDermott J.E., Jacobs J.M., Merrill N.J., Mitchell H.D., Arshad O.A., McClure R., Teeguarden J., Gajula R.P., Porter K.I., Satterfield B.C. (2024). Molecular-Level Dysregulation of Insulin Pathways and Inflammatory Processes in Peripheral Blood Mononuclear Cells by Circadian Misalignment. J. Proteome Res..

[6] Lyu J., Lee K., Jung S., Park Y.J. (2024). Associations of meal timing and sleep duration with incidence of obesity: A prospective cohort study. J. Nutr. Health Aging.

[7] Tian H., Zhao X., Zhang Y., Xia Z. (2024). Research progress of circadian rhythm in cardiovascular disease: A bibliometric study from 2002 to 2022. Heliyon.

[8] Russell K.L., Rodman H.R., Pak V.M. (2023). Sleep insufficiency, circadian rhythms, and metabolomics: The connection between metabolic and sleep disorders. Sleep. Breath..

[9] Gu W., Han T., Sun C. (2023). Association of 24 h Behavior Rhythm with Non-Alcoholic Fatty Liver Disease among American Adults with Overweight/Obesity. Nutrients.

[10] Zhang T., Cheng P., Ma X., Yu X. (2024). Influence of circadian rhythm and sleep schedules on depressive symptoms among adolescents in China. Int. J. Environ. Health Res..

[11] Bima A., Eldakhakhny B., Nuwaylati D., Alnami A., Ajabnoor M., Elsamanoudy A. (2021). The Interplay of Vitamin D Deficiency and Cellular Senescence in the Pathogenesis of Obesity-Related Co-Morbidities. Nutrients.

[12] Gao Y.X., Kou C. (2023). The Associations of Vitamin D Level with Metabolic Syndrome and Its Components among Adult Population: Evidence from National Health and Nutrition Examination Survey 2017–2018. Metab. Syndr. Relat. Disord..

[13] Xia Y., Yu Y., Zhao Y., Deng Z., Zhang L., Liang G. (2023). Insight into the Interaction Mechanism of Vitamin D against Metabolic Syndrome: A Meta-Analysis and In Silico Study. Foods.

[14] Loh H.H., Lim Q.H., Kang W.H., Yee A., Yong M.C., Sukor N. (2023). Obstructive sleep apnea and vitamin D: An updated systematic review and meta-analysis. Hormones.

[15] Jiang J., Tan H., Xia Z., Li J., Zhou S., Huang T. (2024). Serum vitamin D concentrations and sleep disorders: Insights from NHANES 2011–2016 and Mendelian Randomization analysis. Sleep. Breath..

[16] Al-Qahtani F.S., Alshaikh A.A., Alfaifi S.H. (2024). The Association between Vitamin D Deficiency and the Level of Fasting C Peptide Among Patients with Uncontrolled Type 2 Diabetes Mellitus: A Retrospective Cohort Study. Cureus.

[17] Costanzo S., De Curtis A., Di Castelnuovo A., Persichillo M., Bonaccio M., Pounis G., Cerletti C., Donati M.B., de Gaetano G., Iacoviello L. (2018). Serum vitamin D deficiency and risk of hospitalization for heart failure: Prospective results from the Moli-sani study. Nutr. Metab. Cardiovasc. Dis..

[18] Szymczak-Pajor I., Drzewoski J., Śliwińska A. (2020). The Molecular Mechanisms by Which Vitamin D Prevents Insulin Resistance and Associated Disorders. Int. J. Mol. Sci..

[19] Gilon P., Chae H.Y., Rutter G.A., Ravier M.A. (2014). Calcium signaling in pancreatic β-cells in health and in Type 2 diabetes. Cell Calcium.

[20] Gholamzad A., Khakpour N., Kabipour T., Gholamzad M. (2023). Association between serum vitamin D levels and lipid profiles: A cross-sectional analysis. Sci. Rep..

[21] Johnson C.L., Paulose-Ram R., Ogden C.L., Carroll M.D., Kruszon-Moran D., Dohrmann S.M., Curtin L.R. (2013). National Health and Nutrition Examination Survey: Analytic Guidelines, 1999–2010.

[22] Ross A.C., Taylor C.L., Yaktine A.L., Del Valle H.B., Institute of Medicine Committee to Review Dietary Reference Intakes for Vitamin D and Calcium (2011). The National Academies Collection: Reports funded by National Institutes of Health. Dietary Reference Intakes for Calcium and Vitamin D.

[23] Kroenke K., Spitzer R.L., Williams J.B. (2001). The PHQ-9: Validity of a brief depression severity measure. J. Gen. Intern. Med..

[24] Alberti K.G., Eckel R.H., Grundy S.M., Zimmet P.Z., Cleeman J.I., Donato K.A., Fruchart J.C., James W.P., Loria C.M., Smith S.C. (2009). Harmonizing the metabolic syndrome: A joint interim statement of the International Diabetes Federation Task Force on Epidemiology and Prevention; National Heart, Lung, and Blood Institute; American Heart Association; World Heart Federation; International Atherosclerosis Society; and International Association for the Study of Obesity. Circulation.

[25] Drinking Levels Defined. Overview of Alcohol Consumption 2023. https://www.niaaa.nih.gov/alcohol-health/overview-alcohol-consumption/moderate-binge-drinking.

[26] Developing the Healthy Eating Index. The Healthy Eating Index 19 December 2023. https://epi.grants.cancer.gov/hei/developing.html#:~:text=For%20the%20first%20time%2C%20the,2%20and%20older%3B%20and%20the.

[27] Liang J., Huang S., Jiang N., Kakaer A., Chen Y., Liu M., Pu Y., Huang S., Pu X., Zhao Y. (2023). Association Between Joint Physical Activity and Dietary Quality and Lower Risk of Depression Symptoms in US Adults: Cross-sectional NHANES Study. JMIR Public Health Surveill..

[28] Lin Z.H., Xin Y.N., Dong Q.J., Wang Q., Jiang X.J., Zhan S.H., Sun Y., Xuan S.Y. (2011). Performance of the aspartate aminotransferase-to-platelet ratio index for the staging of hepatitis C-related fibrosis: An updated meta-analysis. Hepatology.

[29] Theik N.W.Y., Raji O.E., Shenwai P., Shah R., Kalluri S.R., Bhutta T.H., Hannoodee H., Al Khalili M., Khan S. (2021). Relationship and Effects of Vitamin D on Metabolic Syndrome: A Systematic Review. Cureus.

[30] Muscogiuri G., Barrea L., Scannapieco M., Di Somma C., Scacchi M., Aimaretti G., Savastano S., Colao A., Marzullo P. (2019). The lullaby of the sun: The role of vitamin D in sleep disturbance. Sleep. Med..

[31] Al-Shawwa B., Ehsan Z., Ingram D.G. (2020). Vitamin D and sleep in children. J. Clin. Sleep. Med..

[32] Anglin R.E., Samaan Z., Walter S.D., McDonald S.D. (2013). Vitamin D deficiency and depression in adults: Systematic review and meta-analysis. Br. J. Psychiatry.

[33] Bennouar S., Cherif A.B., Kessira A., Bennouar D.E., Abdi S. (2021). Association and interaction between vitamin D level and metabolic syndrome for non-alcoholic fatty liver disease. J. Diabetes Metab. Disord..

[34] Heo N.J., Park H.E., Yoon J.W., Kwak M.S., Yang J.I., Chung S.J., Yim J.Y., Chung G.E. (2021). The Association between Vitamin D and Nonalcoholic Fatty Liver Disease Assessed by Controlled Attenuation Parameter. J. Clin. Med..

[35] Romano F., Muscogiuri G., Di Benedetto E., Zhukouskaya V.V., Barrea L., Savastano S., Colao A., Di Somma C. (2020). Vitamin D and Sleep Regulation: Is there a Role for Vitamin D?. Curr. Pharm. Des..

[36] Maroufi N.F., Pezeshgi P., Mortezania Z., Pourmohammad P., Eftekhari R., Moradzadeh M., Vahedian V., Nouri M. (2020). Association between vitamin D deficiency and prevalence of metabolic syndrome in female population: A systematic review. Horm. Mol. Biol. Clin. Investig..

[37] Huang X., Yang Y., Jiang Y., Zhou Z., Zhang J. (2023). Association between vitamin D deficiency and lipid profiles in overweight and obese adults: A systematic review and meta-analysis. BMC Public Health.

[38] Mutt S.J., Jokelainen J., Sebert S., Auvinen J., Järvelin M.-R., Keinänen-Kiukaanniemi S., Herzig K.-H. (2019). Vitamin D Status and Components of Metabolic Syndrome in Older Subjects from Northern Finland (Latitude 65° North). Nutrients.

[39] Smolensky M.H., Sackett-Lundeen L.L., Portaluppi F. (2015). Nocturnal light pollution and underexposure to daytime sunlight: Complementary mechanisms of circadian disruption and related diseases. Chronobiol. Int..

[40] Chang A.M., Scheer F.A., Czeisler C.A. (2011). The human circadian system adapts to prior photic history. J. Physiol..

[41] Gutierrez-Monreal M.A., Cuevas-Diaz Duran R., Moreno-Cuevas J.E., Scott S.P. (2014). A role for 1α,25-dihydroxyvitamin d3 in the expression of circadian genes. J. Biol. Rhythm..

