# Supplementary material for: Association between Serum Vitamin D Status and Circadian Syndrome: A Cross-Sectional Study
Source: Nutrients. 2024 Jul 2;16(13):2111. doi: 10.3390/nu16132111 (PMC11243086; doi:10.3390/nu16132111)
Supplement: Supplementary file 1 [file nutrients-16-02111-s001.zip › Supplementary - CircS Vitamin D.pdf]

Supplementary Material

Association Between Serum Vitamin D Status and Circadian Syndrome: A Cross-Sectional Study

Ahmed Arabi[1,†], Dima Nasrallah[1,†], Sara Mohsen[1], Lana Abugharbieh[1], Dana Al-Hashimi[1], Shaikha AlMass[1], Shahd Albasti[1], Saeed A Al-Ajmi[1], Muhammad Naseem Khan[2,*], Susu M Zughaier[3,*]

Correspondence: (M.N. Khan) naseem@qu.edu.qa; Tel.: +974-4403-7839, (S.M. Zughaier) szughaier@qu.edu.qa; Tel.: +974-4403-7859

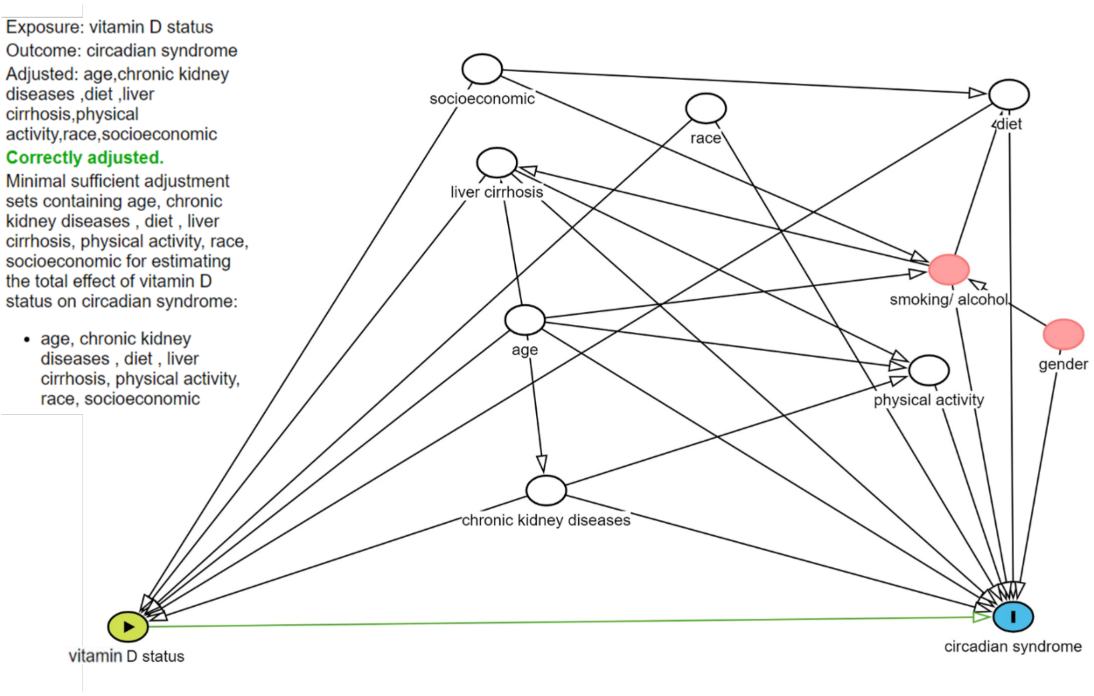

Figure S1. Directed Acyclic Graph (DAG) showing the association between vitamin D status with circadian syndrome and different covariates.

Table S1. Association between serum vitamin D levels and obesity (n = 6,976)[1].

Exposure	Categories	Obesity Odds Ratio	*P*-value	95% CI
**Vitamin D Status**				
	Adequacy	1		
	Inadequacy	1.33	<0.001	(1.17, 1.51)
	Deficiency	1.37	0.002	(1.12, 1.68)

[1] Model adjusted for age, poverty income ratio, education level, diet quality, physical activity, and history of bariatric surgery.

Table S2. Association between serum vitamin D levels and short sleep (n = 28,850)[1].

Exposure	Categories	Short Sleep Odds Ratio	*P*-value	95% CI
**Vitamin D Status**				
	Adequacy	1		
	Inadequacy	1.43	<0.001	(1.32, 1.54)
	Deficiency	1.97	<0.001	(1.76, 2.19)

[1] Model adjusted for age, obesity, liver cirrhosis, and chronic kidney disease.

Table S3. Association between serum vitamin D levels and elevated FPG (n = 17,078)[1].

Exposure	Categories	Elevated FPG Odds Ratio	*P*-value	95% CI
**Vitamin D Status**				
	Adequacy	1		
	Inadequacy	1.20	<0.001	(1.10, 1.30)
	Deficiency	1.21	0.005	(1.06, 1.38)

[1] Model adjusted for age, race, obesity, and liver cirrhosis.

Table S4. Association between serum vitamin D levels and low HDL (n = 25,134)[1].

Exposure	Categories	Low HDL Odds Ratio	*P*-value	95% CI
**Vitamin D Status**				
	Adequacy	1		
	Inadequacy	1.31	<0.001	(1.23, 1.40)
	Deficiency	1.44	<0.001	(1.29, 1.60)

[1] Model adjusted for age, race, obesity, diet quality, and liver cirrhosis.

Table S5. Association between serum vitamin D levels and elevated TG (n = 12,396)[1].

Exposure	Categories	Elevated TG Odds Ratio	*P*-value	95% CI
**Vitamin D Status**				
	Adequacy	1		
	Inadequacy	1.07	0.233	(0.96, 1.18)
	Deficiency	1.10	0.262	(0.93, 1.31)

[1] Model adjusted for age, race, obesity, diet quality, and liver cirrhosis.

Table S6. Association between serum vitamin D levels and elevated blood pressure (EBP) (n = 10,043)[1].

Exposure	Categories	EBP Odds Ratio	*P*-value	95% CI
**Vitamin D Status**				
	Adequacy	1		
	Inadequacy	1.07	0.264	(0.95, 1.21)
	Deficiency	1.25	0.025	(1.03, 1.51)

[1] Model adjusted for age, race, diet quality, dyslipidemia, and chronic kidney disease.

Table S7. Association between serum vitamin D levels and NAFLD (n = 8,703)[1].

Exposure	Categories	NAFLD Odds Ratio	*P*-value	95% CI
**Vitamin D Status**				
	Adequacy	1		
	Inadequacy	1.12	0.466	(0.83, 1.50)
	Deficiency	1.65	0.021	(1.08, 2.53)

[1] Model adjusted for age, race, diet quality, and dyslipidemia.

Table S8. Association between serum vitamin D levels and Depression (n = 21,045)[1].

Exposure	Categories	Depression Odds Ratio	*P*-value	95% CI
**Vitamin D Status**				
	Adequacy	1		
	Inadequacy	1.03	0.609	(0.91, 1.17)
	Deficiency	1.25	0.012	(1.05, 1.49)

[1] Model adjusted for age, race, poverty income ratio, education level, diet quality, obesity, liver cirrhosis, and chronic kidney disease.
